# (*E*)-1-[2-(4-Fluoro-2-nitro­styr­yl)-1-phenyl­sulfonyl-1*H*-indol-3-yl]propan-1-one

**DOI:** 10.1107/S1600536813030961

**Published:** 2013-11-16

**Authors:** M. Umadevi, V. Saravanan, R. Yamuna, A. K. Mohanakrishnan, G. Chakkaravarthi

**Affiliations:** a Research Scholar (Chemistry), Bharathiyar University, Coimbatore 641 046, Tamilnadu, India; b Department of Organic Chemistry, University of Madras, Guindy Campus, Chennai 600 025, India; c Department of Sciences, Chemistry and Materials Research Lab, Amrita Vishwa Vidyapeetham University, Ettimadai, Coimbatore 641 112, India; d Department of Physics, CPCL Polytechnic College, Chennai 600 068, India

## Abstract

In the title compound, C_25_H_19_FN_2_O_5_S, the substituted phenyl ring makes a dihedral angle of 12.26 (9)° with the indole ring system. The nitro group is twisted at an angle of 26.92 (8)° out of the plane of the ring to which it is attached. The mol­ecular structure is stabilized by weak C—H⋯O hydrogen bonds. In the crystal, weak C—H⋯O, C—H⋯F and π–π [centroid–centroid distance = 3.6645 (11) Å] inter­actions link the mol­ecules, forming a three-dimensional network.

## Related literature

For the biological activity of indole derivatives, see: Pomarnacka & Kozlarska-Kedra (2003 ▶); Srivastava *et al.* (2011 ▶). For related structures, see: Chakkaravarthi *et al.* (2008 ▶, 2010 ▶). For details of the configuration at the S atom, see: Bassindale (1984 ▶). For details of N-atom hybridization, see: Beddoes *et al.* (1986 ▶).
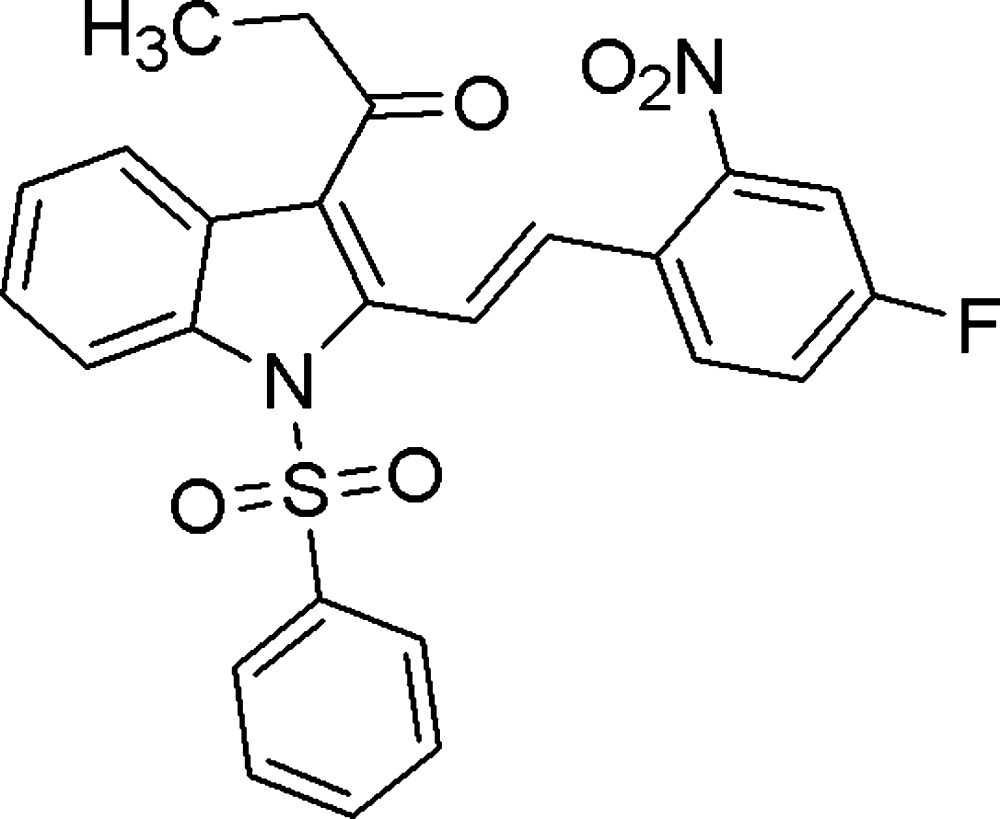


## Experimental

### 

#### Crystal data

C_25_H_19_FN_2_O_5_S
*M* *_r_* = 478.48
Triclinic, 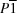

*a* = 8.2615 (3) Å
*b* = 10.7624 (5) Å
*c* = 13.2432 (6) Å
α = 68.606 (2)°
β = 80.554 (3)°
γ = 81.012 (2)°
*V* = 1075.53 (8) Å^3^
*Z* = 2
Mo *K*α radiation
μ = 0.20 mm^−1^
*T* = 295 K
0.30 × 0.24 × 0.20 mm

#### Data collection

Bruker APEXII diffractometer
Absorption correction: multi-scan (*SADABS*; Sheldrick, 1996 ▶) *T*_min_ = 0.942, *T*_max_ = 0.961
27388 measured reflections
8185 independent reflections
5506 reflections with *I* > 2σ(*I*)
*R*_int_ = 0.028

#### Refinement

*R*[*F* ^2^ > 2σ(*F* ^2^)] = 0.055
*wR*(*F* ^2^) = 0.176
*S* = 1.03
8185 reflections
308 parameters
H-atom parameters constrained
Δρ_max_ = 0.96 e Å^−3^
Δρ_min_ = −0.61 e Å^−3^

### 

Data collection: *APEX2* (Bruker, 2004 ▶); cell refinement: *SAINT* (Bruker, 2004 ▶); data reduction: *SAINT*; program(s) used to solve structure: *SHELXS97* (Sheldrick, 2008 ▶); program(s) used to refine structure: *SHELXL97* (Sheldrick, 2008 ▶); molecular graphics: *PLATON* (Spek, 2009 ▶); software used to prepare material for publication: *SHELXL97*.

## Supplementary Material

Crystal structure: contains datablock(s) I, global. DOI: 10.1107/S1600536813030961/bt6945sup1.cif

Structure factors: contains datablock(s) I. DOI: 10.1107/S1600536813030961/bt6945Isup2.hkl

Supplementary material file. DOI: 10.1107/S1600536813030961/bt6945Isup3.cml

Additional supplementary materials:  crystallographic information; 3D view; checkCIF report

## Figures and Tables

**Table 1:** Hydrogen-bond geometry (Å, °)

*D*—H⋯*A*	*D*—H	H⋯*A*	*D*⋯*A*	*D*—H⋯*A*
C8—H8⋯O1	0.93	2.33	2.913 (3)	120
C11—H11⋯O3	0.93	2.40	2.905 (3)	114
C16—H16*A*⋯F1^i^	0.97	2.54	3.192 (2)	124
C22—H22⋯O4^ii^	0.93	2.52	3.438 (2)	170

Symmetry codes: (i) 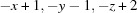; (ii) 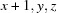.

**Fig. 1.**
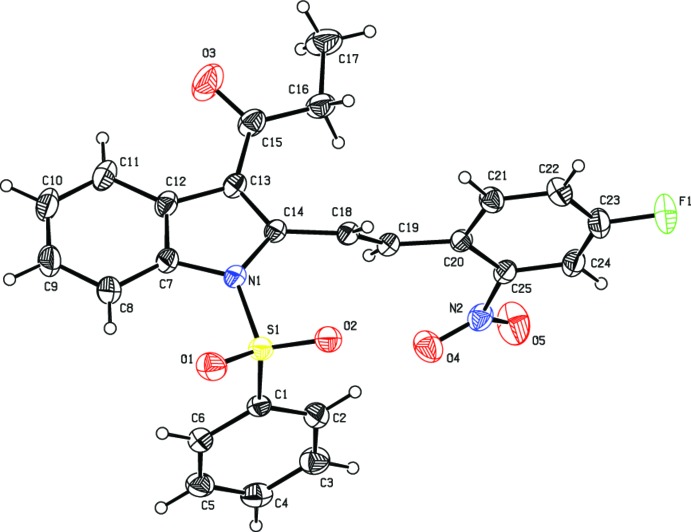
The molecular structure of the title compound, with atom labels and 30% probability displacement ellipsoids for non-H atoms.

**Fig. 2.**
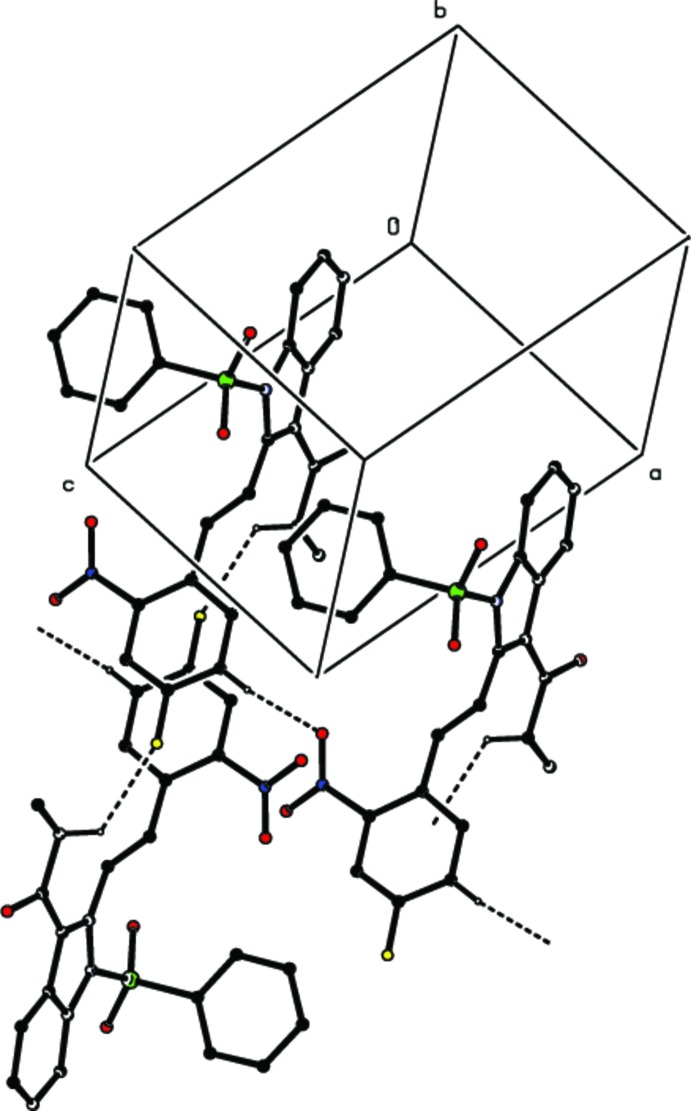
The packing of the title compound, view onto the ac plane. Intermolecular hydrogen bonds are shown as dashed lines. H atoms not involving hydrogen bonding have been omitted.

## supplementary crystallographic information

### 1. Comment

Indole derivatives are known to exhibit antimicrobial, antibiotic, analgesic, anticancer and anti-HIV (Pomarnacka & Kozlarska-Kedra, 2003; Srivastava *et al.*, 2011) activities. In continuation of our studies on indole derivatives, we determined the crystal structure of the title compound (I). The geometric parameters of (I) (Fig. 1) are agree well with the reported structures (Chakkaravarthi *et al.*, 2008; 2010).

Due to Thorpe-Ignold effect (Bassindale, 1984), bond angles around atom S1 show significant deviation from ideal tetrahedral value, with significant deviations in angles O1—S1—O2 [120.42 (8)°] and N1—S1—C1 [104.66 (7)°]. The phenyl ring (C1—C6) makes the dihedral angle of 85.05 (8)° with the indole ring system. The phenyl ring (C1—C6) and the benzene ring (C20—C25) are inclined at an angle of 12.26 (9)°. The nitro group is twisted at an angle of 26.92 (8)° with the attached benzene ring (C20—C25). The sum of the bond angles around N1 (358.26°) indicates the *sp*^2^ hybridization of N1 atom (Beddoes *et al.*, 1986).

The molecular structure is stabilized by weak intramolecular C—H···O hydrogen bonds (Table 1). The crystal structure exhibit weak intermolecular C—H···O, C—H···F (Table 1 & Fig. 2) and π···π [*Cg*4···*Cg*4^i^ = 3.6645 (11) Å; (i) 1 - *x*, -1 - *y*, 2 - *z*; *Cg*4 is the centroid of the ring (C20—C25)] interactions.

### 2. Experimental

A solution of 1-(2-(bromomethyl)-1-(phenylsulfonyl)-1*H*-indol-3-yl) propan-1-one (5 g, 12.31 mmol) and triphenylphosphine (3.5 g, 13.54 mmol) in dry THF (100 ml) was refluxed for 6 h. After consumption of the starting material, the solvent was removed under vacuo and the solid was washed with diethyl ether to give the phosphonium salt. Then, the mixture of phosphonium salt (8 g, 11.97 mmol), 4-fluoro-2-nitrobenzaldehyde (2.24 g, 13.17 mmol) and K_2_CO_3_ (3.30 g, 23.95 mmol) in DCM (70 ml) was stirred at room temperature for 22 h. After completion of the reaction (monitored by TLC), it was diluted using DCM (30 ml), washed with water (2 *x* 100 ml) and dried (Na_2_SO_4_). Removal of solvent *in vacuo* followed by trituration of the crude product with MeOH (20 ml) afforded the title compound suitable for X-ray diffraction quality.

### 3. Refinement

The H atoms were positioned geometrically and refined using a riding model with C—H = 0.93–0.97 Å, and with *U*_iso_(H) = 1.2*U*_eq_(C) (or) *U*_iso_(H) = 1.5*U*_eq_(C).

### Crystal data

**Table:**

C_25_H_19_FN_2_O_5_S	*Z* = 2
*M**_r_* = 478.48	*F*(000) = 496
Triclinic, *P*1	*D*_x_ = 1.477 Mg m^−^^3^
Hall symbol: -P 1	Mo *K*α radiation, λ = 0.71073 Å
*a* = 8.2615 (3) Å	Cell parameters from 5619 reflections
*b* = 10.7624 (5) Å	θ = 2.1–31.1°
*c* = 13.2432 (6) Å	µ = 0.20 mm^−^^1^
α = 68.606 (2)°	*T* = 295 K
β = 80.554 (3)°	Block, colourless
γ = 81.012 (2)°	0.30 × 0.24 × 0.20 mm
*V* = 1075.53 (8) Å^3^	

### Data collection

**Table:**

Bruker APEXII diffractometer	8185 independent reflections
Radiation source: fine-focus sealed tube	5506 reflections with *I* > 2σ(*I*)
Graphite monochromator	*R*_int_ = 0.028
ω and φ scan	θ_max_ = 35.0°, θ_min_ = 1.7°
Absorption correction: multi-scan (*SADABS*; Sheldrick, 1996)	*h* = −12→12
*T*_min_ = 0.942, *T*_max_ = 0.961	*k* = −16→17
27388 measured reflections	*l* = −20→21

### Refinement

**Table:**

Refinement on *F*^2^	Primary atom site location: structure-invariant direct methods
Least-squares matrix: full	Secondary atom site location: difference Fourier map
*R*[*F*^2^ > 2σ(*F*^2^)] = 0.055	Hydrogen site location: inferred from neighbouring sites
*wR*(*F*^2^) = 0.176	H-atom parameters constrained
*S* = 1.03	*w* = 1/[σ^2^(*F*_o_^2^) + (0.089*P*)^2^ + 0.2703*P*] where *P* = (*F*_o_^2^ + 2*F*_c_^2^)/3
8185 reflections	(Δ/σ)_max_ < 0.001
308 parameters	Δρ_max_ = 0.96 e Å^−^^3^
0 restraints	Δρ_min_ = −0.61 e Å^−^^3^

### Special details

**Table:**

Geometry. All e.s.d.'s (except the e.s.d. in the dihedral angle between two l.s. planes) are estimated using the full covariance matrix. The cell e.s.d.'s are taken into account individually in the estimation of e.s.d.'s in distances, angles and torsion angles; correlations between e.s.d.'s in cell parameters are only used when they are defined by crystal symmetry. An approximate (isotropic) treatment of cell e.s.d.'s is used for estimating e.s.d.'s involving l.s. planes.
Refinement. Refinement of *F*^2^ against ALL reflections. The weighted *R*-factor *wR* and goodness of fit *S* are based on *F*^2^, conventional *R*-factors *R* are based on *F*, with *F* set to zero for negative *F*^2^. The threshold expression of *F*^2^ > σ(*F*^2^) is used only for calculating *R*-factors(gt) *etc*. and is not relevant to the choice of reflections for refinement. *R*-factors based on *F*^2^ are statistically about twice as large as those based on *F*, and *R*- factors based on ALL data will be even larger.

### Fractional atomic coordinates and isotropic or equivalent isotropic displacement parameters (Å^2^)

**Table:**

	*x*	*y*	*z*	*U*_iso_*/*U*_eq_	
C1	−0.05337 (18)	0.17906 (15)	0.76350 (12)	0.0387 (3)	
C2	−0.0663 (2)	0.06833 (18)	0.85866 (14)	0.0476 (4)	
H2	0.0275	0.0203	0.8901	0.057*	
C3	−0.2214 (2)	0.0306 (2)	0.90598 (16)	0.0563 (4)	
H3	−0.2324	−0.0430	0.9702	0.068*	
C4	−0.3602 (2)	0.1021 (2)	0.85825 (16)	0.0540 (4)	
H4	−0.4641	0.0762	0.8907	0.065*	
C5	−0.3457 (2)	0.2109 (2)	0.76326 (16)	0.0548 (4)	
H5	−0.4397	0.2576	0.7314	0.066*	
C6	−0.1920 (2)	0.25156 (19)	0.71465 (14)	0.0489 (4)	
H6	−0.1817	0.3257	0.6507	0.059*	
C7	0.1475 (2)	0.19323 (17)	0.50807 (12)	0.0439 (3)	
C8	0.0691 (3)	0.3166 (2)	0.44803 (15)	0.0596 (5)	
H8	0.0385	0.3851	0.4765	0.072*	
C9	0.0389 (3)	0.3323 (2)	0.34425 (16)	0.0668 (6)	
H9	−0.0130	0.4135	0.3020	0.080*	
C10	0.0836 (3)	0.2308 (2)	0.30181 (15)	0.0637 (5)	
H10	0.0627	0.2457	0.2312	0.076*	
C11	0.1583 (2)	0.1080 (2)	0.36119 (13)	0.0544 (4)	
H11	0.1858	0.0397	0.3322	0.065*	
C12	0.1919 (2)	0.08841 (17)	0.46722 (12)	0.0429 (3)	
C13	0.27195 (19)	−0.02364 (15)	0.54893 (12)	0.0403 (3)	
C14	0.27322 (18)	0.01380 (14)	0.63737 (11)	0.0367 (3)	
C15	0.3524 (3)	−0.14616 (18)	0.52664 (14)	0.0522 (4)	
C16	0.4209 (3)	−0.26746 (18)	0.61156 (15)	0.0563 (4)	
H16A	0.3365	−0.2957	0.6730	0.068*	
H16B	0.5119	−0.2449	0.6373	0.068*	
C17	0.4816 (4)	−0.3835 (2)	0.5708 (2)	0.0767 (7)	
H17A	0.3896	−0.4137	0.5537	0.115*	
H17B	0.5345	−0.4558	0.6265	0.115*	
H17C	0.5589	−0.3542	0.5065	0.115*	
C18	0.34816 (19)	−0.05675 (15)	0.73895 (11)	0.0380 (3)	
H18	0.4365	−0.0220	0.7510	0.046*	
C19	0.29602 (19)	−0.16804 (15)	0.81477 (11)	0.0380 (3)	
H19	0.2011	−0.1976	0.8059	0.046*	
C20	0.37971 (18)	−0.24690 (14)	0.91153 (11)	0.0360 (3)	
C21	0.5492 (2)	−0.24341 (16)	0.90850 (14)	0.0454 (3)	
H21	0.6053	−0.1874	0.8457	0.054*	
C22	0.6361 (2)	−0.31934 (18)	0.99463 (15)	0.0510 (4)	
H22	0.7478	−0.3125	0.9913	0.061*	
C23	0.5542 (2)	−0.40558 (17)	1.08570 (15)	0.0511 (4)	
C24	0.3899 (2)	−0.41643 (16)	1.09468 (13)	0.0462 (4)	
H24	0.3370	−0.4760	1.1569	0.055*	
C25	0.30441 (19)	−0.33571 (15)	1.00810 (11)	0.0384 (3)	
N1	0.20107 (17)	0.14790 (13)	0.61298 (10)	0.0410 (3)	
N2	0.12683 (19)	−0.34714 (16)	1.02366 (11)	0.0489 (3)	
O1	0.12824 (18)	0.36656 (12)	0.64011 (11)	0.0580 (3)	
O2	0.25410 (15)	0.17470 (13)	0.78405 (10)	0.0492 (3)	
O3	0.3708 (3)	−0.1422 (2)	0.43278 (14)	0.1065 (8)	
O4	0.03576 (17)	−0.25160 (16)	0.97436 (13)	0.0684 (4)	
O5	0.0783 (2)	−0.45267 (19)	1.08539 (15)	0.0913 (6)	
S1	0.14288 (5)	0.22764 (4)	0.70332 (3)	0.04104 (11)	
F1	0.64046 (18)	−0.48260 (14)	1.16881 (11)	0.0778 (4)	

### Atomic displacement parameters (Å^2^)

**Table:**

	*U*^11^	*U*^22^	*U*^33^	*U*^12^	*U*^13^	*U*^23^
C1	0.0383 (7)	0.0433 (7)	0.0372 (7)	−0.0005 (5)	−0.0030 (5)	−0.0194 (6)
C2	0.0446 (8)	0.0492 (9)	0.0453 (8)	−0.0023 (6)	−0.0044 (6)	−0.0135 (7)
C3	0.0536 (10)	0.0562 (10)	0.0553 (10)	−0.0114 (8)	0.0045 (8)	−0.0171 (8)
C4	0.0426 (8)	0.0676 (11)	0.0620 (11)	−0.0103 (8)	0.0031 (7)	−0.0366 (9)
C5	0.0401 (8)	0.0756 (12)	0.0585 (10)	0.0042 (8)	−0.0096 (7)	−0.0372 (10)
C6	0.0465 (9)	0.0570 (10)	0.0430 (8)	0.0034 (7)	−0.0073 (6)	−0.0199 (7)
C7	0.0439 (8)	0.0475 (8)	0.0315 (6)	−0.0027 (6)	−0.0015 (6)	−0.0055 (6)
C8	0.0646 (11)	0.0569 (11)	0.0416 (9)	0.0094 (9)	−0.0054 (8)	−0.0053 (8)
C9	0.0628 (12)	0.0732 (13)	0.0416 (9)	0.0056 (10)	−0.0100 (8)	0.0033 (9)
C10	0.0587 (11)	0.0875 (15)	0.0335 (8)	−0.0111 (10)	−0.0098 (7)	−0.0047 (9)
C11	0.0584 (10)	0.0704 (12)	0.0337 (7)	−0.0154 (9)	−0.0069 (7)	−0.0131 (7)
C12	0.0430 (8)	0.0509 (8)	0.0304 (6)	−0.0113 (6)	−0.0013 (5)	−0.0078 (6)
C13	0.0456 (8)	0.0412 (7)	0.0322 (6)	−0.0095 (6)	−0.0010 (5)	−0.0100 (5)
C14	0.0390 (7)	0.0370 (7)	0.0303 (6)	−0.0049 (5)	−0.0004 (5)	−0.0082 (5)
C15	0.0694 (11)	0.0485 (9)	0.0438 (8)	−0.0094 (8)	−0.0045 (8)	−0.0216 (7)
C16	0.0739 (12)	0.0455 (9)	0.0468 (9)	0.0002 (8)	0.0042 (8)	−0.0202 (7)
C17	0.1053 (19)	0.0522 (11)	0.0682 (13)	−0.0006 (11)	0.0154 (13)	−0.0298 (10)
C18	0.0422 (7)	0.0379 (7)	0.0326 (6)	−0.0020 (5)	−0.0038 (5)	−0.0118 (5)
C19	0.0397 (7)	0.0407 (7)	0.0315 (6)	−0.0033 (5)	−0.0026 (5)	−0.0110 (5)
C20	0.0412 (7)	0.0340 (6)	0.0318 (6)	−0.0024 (5)	−0.0033 (5)	−0.0116 (5)
C21	0.0423 (8)	0.0421 (8)	0.0453 (8)	−0.0035 (6)	−0.0041 (6)	−0.0085 (6)
C22	0.0444 (8)	0.0480 (9)	0.0570 (10)	−0.0009 (7)	−0.0135 (7)	−0.0127 (7)
C23	0.0628 (11)	0.0431 (8)	0.0458 (8)	0.0028 (7)	−0.0217 (8)	−0.0105 (7)
C24	0.0606 (10)	0.0419 (8)	0.0334 (7)	−0.0055 (7)	−0.0074 (6)	−0.0089 (6)
C25	0.0460 (8)	0.0367 (7)	0.0322 (6)	−0.0055 (5)	−0.0031 (5)	−0.0119 (5)
N1	0.0473 (7)	0.0399 (6)	0.0302 (5)	0.0019 (5)	−0.0018 (5)	−0.0093 (5)
N2	0.0490 (8)	0.0547 (8)	0.0382 (7)	−0.0129 (6)	0.0003 (6)	−0.0096 (6)
O1	0.0655 (8)	0.0389 (6)	0.0634 (8)	−0.0044 (5)	0.0000 (6)	−0.0142 (6)
O2	0.0431 (6)	0.0580 (7)	0.0523 (7)	−0.0025 (5)	−0.0092 (5)	−0.0258 (6)
O3	0.187 (2)	0.0818 (12)	0.0574 (9)	0.0227 (13)	−0.0319 (12)	−0.0396 (9)
O4	0.0455 (7)	0.0704 (9)	0.0690 (9)	−0.0035 (6)	−0.0025 (6)	−0.0031 (7)
O5	0.0707 (10)	0.0820 (11)	0.0877 (12)	−0.0339 (9)	−0.0065 (9)	0.0192 (9)
S1	0.0421 (2)	0.03958 (19)	0.0412 (2)	−0.00283 (14)	−0.00230 (14)	−0.01553 (15)
F1	0.0838 (9)	0.0747 (8)	0.0621 (7)	−0.0007 (7)	−0.0385 (7)	0.0009 (6)

### Geometric parameters (Å, º)

**Table:**

C1—C2	1.385 (2)	C15—O3	1.213 (2)
C1—C6	1.391 (2)	C15—C16	1.482 (3)
C1—S1	1.7532 (16)	C16—C17	1.520 (3)
C2—C3	1.383 (3)	C16—H16A	0.9700
C2—H2	0.9300	C16—H16B	0.9700
C3—C4	1.383 (3)	C17—H17A	0.9600
C3—H3	0.9300	C17—H17B	0.9600
C4—C5	1.374 (3)	C17—H17C	0.9600
C4—H4	0.9300	C18—C19	1.330 (2)
C5—C6	1.385 (3)	C18—H18	0.9300
C5—H5	0.9300	C19—C20	1.4678 (19)
C6—H6	0.9300	C19—H19	0.9300
C7—C8	1.394 (2)	C20—C21	1.401 (2)
C7—C12	1.397 (2)	C20—C25	1.403 (2)
C7—N1	1.4155 (19)	C21—C22	1.375 (2)
C8—C9	1.382 (3)	C21—H21	0.9300
C8—H8	0.9300	C22—C23	1.373 (3)
C9—C10	1.379 (3)	C22—H22	0.9300
C9—H9	0.9300	C23—F1	1.3465 (19)
C10—C11	1.375 (3)	C23—C24	1.363 (3)
C10—H10	0.9300	C24—C25	1.384 (2)
C11—C12	1.410 (2)	C24—H24	0.9300
C11—H11	0.9300	C25—N2	1.466 (2)
C12—C13	1.448 (2)	N1—S1	1.6824 (13)
C13—C14	1.373 (2)	N2—O5	1.215 (2)
C13—C15	1.488 (2)	N2—O4	1.216 (2)
C14—N1	1.4131 (19)	O1—S1	1.4193 (13)
C14—C18	1.467 (2)	O2—S1	1.4235 (13)
			
C2—C1—C6	121.56 (16)	C17—C16—H16A	109.0
C2—C1—S1	118.92 (12)	C15—C16—H16B	109.0
C6—C1—S1	119.52 (13)	C17—C16—H16B	109.0
C3—C2—C1	118.70 (16)	H16A—C16—H16B	107.8
C3—C2—H2	120.7	C16—C17—H17A	109.5
C1—C2—H2	120.7	C16—C17—H17B	109.5
C2—C3—C4	120.28 (18)	H17A—C17—H17B	109.5
C2—C3—H3	119.9	C16—C17—H17C	109.5
C4—C3—H3	119.9	H17A—C17—H17C	109.5
C5—C4—C3	120.48 (17)	H17B—C17—H17C	109.5
C5—C4—H4	119.8	C19—C18—C14	123.25 (14)
C3—C4—H4	119.8	C19—C18—H18	118.4
C4—C5—C6	120.46 (17)	C14—C18—H18	118.4
C4—C5—H5	119.8	C18—C19—C20	123.78 (14)
C6—C5—H5	119.8	C18—C19—H19	118.1
C5—C6—C1	118.52 (17)	C20—C19—H19	118.1
C5—C6—H6	120.7	C21—C20—C25	115.36 (14)
C1—C6—H6	120.7	C21—C20—C19	119.86 (13)
C8—C7—C12	122.28 (16)	C25—C20—C19	124.64 (14)
C8—C7—N1	130.77 (17)	C22—C21—C20	122.68 (15)
C12—C7—N1	106.93 (13)	C22—C21—H21	118.7
C9—C8—C7	117.0 (2)	C20—C21—H21	118.7
C9—C8—H8	121.5	C23—C22—C21	118.50 (17)
C7—C8—H8	121.5	C23—C22—H22	120.8
C10—C9—C8	121.67 (19)	C21—C22—H22	120.8
C10—C9—H9	119.2	F1—C23—C24	119.04 (17)
C8—C9—H9	119.2	F1—C23—C22	118.45 (17)
C11—C10—C9	121.72 (18)	C24—C23—C22	122.51 (16)
C11—C10—H10	119.1	C23—C24—C25	117.75 (15)
C9—C10—H10	119.1	C23—C24—H24	121.1
C10—C11—C12	118.23 (19)	C25—C24—H24	121.1
C10—C11—H11	120.9	C24—C25—C20	123.15 (15)
C12—C11—H11	120.9	C24—C25—N2	115.60 (14)
C7—C12—C11	119.09 (16)	C20—C25—N2	121.24 (13)
C7—C12—C13	108.19 (13)	C14—N1—C7	108.72 (12)
C11—C12—C13	132.70 (17)	C14—N1—S1	125.70 (10)
C14—C13—C12	107.60 (14)	C7—N1—S1	123.84 (11)
C14—C13—C15	129.90 (15)	O5—N2—O4	123.33 (17)
C12—C13—C15	122.08 (14)	O5—N2—C25	117.83 (16)
C13—C14—N1	108.51 (13)	O4—N2—C25	118.84 (14)
C13—C14—C18	130.55 (14)	O1—S1—O2	120.42 (8)
N1—C14—C18	120.63 (13)	O1—S1—N1	105.64 (7)
O3—C15—C16	119.04 (18)	O2—S1—N1	106.91 (7)
O3—C15—C13	117.57 (18)	O1—S1—C1	109.25 (8)
C16—C15—C13	123.24 (14)	O2—S1—C1	108.80 (7)
C15—C16—C17	113.04 (17)	N1—S1—C1	104.66 (7)
C15—C16—H16A	109.0		
			
C6—C1—C2—C3	−0.6 (3)	C25—C20—C21—C22	−1.3 (2)
S1—C1—C2—C3	179.82 (14)	C19—C20—C21—C22	−177.11 (15)
C1—C2—C3—C4	0.5 (3)	C20—C21—C22—C23	2.4 (3)
C2—C3—C4—C5	0.2 (3)	C21—C22—C23—F1	178.20 (16)
C3—C4—C5—C6	−0.7 (3)	C21—C22—C23—C24	−1.5 (3)
C4—C5—C6—C1	0.6 (3)	F1—C23—C24—C25	179.81 (15)
C2—C1—C6—C5	0.0 (2)	C22—C23—C24—C25	−0.5 (3)
S1—C1—C6—C5	179.63 (13)	C23—C24—C25—C20	1.7 (2)
C12—C7—C8—C9	−1.0 (3)	C23—C24—C25—N2	−177.55 (15)
N1—C7—C8—C9	177.10 (18)	C21—C20—C25—C24	−0.8 (2)
C7—C8—C9—C10	0.2 (3)	C19—C20—C25—C24	174.77 (14)
C8—C9—C10—C11	1.0 (3)	C21—C20—C25—N2	178.40 (14)
C9—C10—C11—C12	−1.3 (3)	C19—C20—C25—N2	−6.0 (2)
C8—C7—C12—C11	0.7 (3)	C13—C14—N1—C7	2.37 (17)
N1—C7—C12—C11	−177.79 (14)	C18—C14—N1—C7	176.59 (13)
C8—C7—C12—C13	179.31 (17)	C13—C14—N1—S1	167.73 (11)
N1—C7—C12—C13	0.79 (17)	C18—C14—N1—S1	−18.1 (2)
C10—C11—C12—C7	0.4 (3)	C8—C7—N1—C14	179.72 (18)
C10—C11—C12—C13	−177.72 (17)	C12—C7—N1—C14	−1.92 (17)
C7—C12—C13—C14	0.66 (17)	C8—C7—N1—S1	14.0 (3)
C11—C12—C13—C14	178.97 (17)	C12—C7—N1—S1	−167.62 (11)
C7—C12—C13—C15	−172.65 (15)	C24—C25—N2—O5	−27.0 (2)
C11—C12—C13—C15	5.7 (3)	C20—C25—N2—O5	153.67 (18)
C12—C13—C14—N1	−1.84 (16)	C24—C25—N2—O4	153.21 (16)
C15—C13—C14—N1	170.76 (16)	C20—C25—N2—O4	−26.1 (2)
C12—C13—C14—C18	−175.29 (15)	C14—N1—S1—O1	161.77 (13)
C15—C13—C14—C18	−2.7 (3)	C7—N1—S1—O1	−34.98 (15)
C14—C13—C15—O3	−161.0 (2)	C14—N1—S1—O2	32.38 (15)
C12—C13—C15—O3	10.7 (3)	C7—N1—S1—O2	−164.37 (13)
C14—C13—C15—C16	14.4 (3)	C14—N1—S1—C1	−82.94 (14)
C12—C13—C15—C16	−173.90 (17)	C7—N1—S1—C1	80.31 (14)
O3—C15—C16—C17	−10.1 (3)	C2—C1—S1—O1	−154.93 (13)
C13—C15—C16—C17	174.49 (19)	C6—C1—S1—O1	25.46 (15)
C13—C14—C18—C19	−67.9 (2)	C2—C1—S1—O2	−21.66 (15)
N1—C14—C18—C19	119.31 (17)	C6—C1—S1—O2	158.73 (12)
C14—C18—C19—C20	173.75 (13)	C2—C1—S1—N1	92.33 (13)
C18—C19—C20—C21	−26.3 (2)	C6—C1—S1—N1	−87.28 (13)
C18—C19—C20—C25	158.25 (15)		

### Hydrogen-bond geometry (Å, º)

**Table:**

*D*—H···*A*	*D*—H	H···*A*	*D*···*A*	*D*—H···*A*
C8—H8···O1	0.93	2.33	2.913 (3)	120
C11—H11···O3	0.93	2.40	2.905 (3)	114
C16—H16*A*···F1^i^	0.97	2.54	3.192 (2)	124
C22—H22···O4^ii^	0.93	2.52	3.438 (2)	170

Symmetry codes: (i) −*x*+1, −*y*−1, −*z*+2; (ii) *x*+1, *y*, *z*.
